# A76 COMBINING TOFACITINIB AND USTEKINUMAB FOR REFRACTORY ULCERATIVE COLITIS: A CASE REPORT

**DOI:** 10.1093/jcag/gwad061.076

**Published:** 2024-02-14

**Authors:** M A Bucheeri, B Alabdulkarim, S Wolman

**Affiliations:** Adult Gastroenterology and Hepatology, University of Toronto, Toronto, ON, Canada; Gastroentertology, University of Toronto, Toronto, ON, Canada; Adult Gastroenterology and Hepatology, University of Toronto, Toronto, ON, Canada

## Abstract

**Background:**

The paradigm for Inflammatory Bowel Disease (IBD) management has shifted towards prioritizing remission of inflammation over symptom control, emphasizing sustained, corticosteroid-free remission at the clinical, endoscopic, and histologic levels. The shift is due to the recognition that uncontrolled inflammation often causes morbidity; after one year of biological therapy, 40% of patients still experience persistent disease activity.

Several approaches have been proposed to address this challenge, including drug level monitoring, dosage titration, antibody screening, and, as a last resort, surgical intervention. Emerging limited studies suggest the potential efficacy of combining two biological agents or a biological agent with small molecules in IBD, contrasting the efficacy and safety concerns encountered with their use in rheumatological diseases.

**Aims:**

To describe a case of successful combination therapy with Tofacitinib and Ustekinumab in refractory Ulcerative Colitis with a review of the current literature.

**Methods:**

This case describes a 35-year-old male patient with severe Ulcerative Colitis refractory to 5-ASA, Azathioprine, Infliximab with Methotrexate, Vedolizumab, Ustekinumab and Tofacitinib. His disease persisted with primary treatment failures requiring hospital admissions and rescuing with parenteral steroids. He was switched back to Ustekinumab with minimal symptom improvement but required repeated steroid courses to achieve control. After deliberations, Tofacitinib was added. This combination achieved remission of his symptoms and inflammatory markers, mainly guided by fecal calprotectin (from 3728 µg/g to 46 µg/g), as well as endoscopic and histologic remission. He is being weaned off the oral budesonide he had been on to augment his response.

**Results:**

After 4 years of combination therapy, he remains in remission with no adverse events or infections.

Reassuringly, Janus Kinase inhibitors (i.e., Tofacitinib) have a short half-life, allowing prompt discontinuation if indicated, especially in the event of infection. Furthermore, biologics such as Vedolizumab, Ustekinumab, and Risankizumab offer minimal systemic immunosuppression and do not appear to raise the risk of infection.

Apart from safety concerns and limited data on the use of combination therapy, significant economic implications exist. However, the attainability of cheaper generic forms of small molecules (e.g., Tofacitinib roughly costs a quarter of the brand price in Ontario, Canada) adds another advantage to their combination use.

**Conclusions:**

Combination therapy with biologics and small molecules is a potential consideration for treating severe or refractory Ulcerative Colitis or IBD. To bridge the knowledge gap regarding combination therapies in IBD, further studies are needed on efficacy, safety, long-term effects, and economic impact.

**Funding Agencies:**

None

